# Internally-Developed Teen Smoking Cessation Programs: Characterizing the Unique Features of Programs Developed by Community-Based Organizations

**DOI:** 10.3390/ijerph6031026

**Published:** 2009-03-10

**Authors:** Kymberle L. Sterling, Susan J. Curry, Sherry Emery, Amy K. Sporer, Robin J. Mermelstein, Michael Berbaum, Brian Flay

**Affiliations:** 1 Institute of Public Health, College of Health and Human Sciences, Georgia State University, 140 Decatur Street, Room 878, Atlanta, Georgia, 30303, USA; 2 College of Public Health, Health Management and Policy, University of Iowa, 200 Hawkins Drive, E220H1 GH, Iowa City, IA 52242, USA; E-mail: sjcurry@iowa.uiowa.edu; 3 Institute for Health Research and Policy, University of Illinois at Chicago, 1747 W. Roosevelt Road, Room 558, Chicago, IL 60608, USA; E-mails: slemery@uic.edu (S.E.); aksporer@uic.edu (A.S.); robinm@uic.edu (R.M.); mberbaum@uic.edu. (M.B.); 4 Department of Public Health, Oregon State University, College of Health and Human Sciences, Oregon State University, Waldo Hall #321, Corvallis, OR 97331, USA; E-mail: brian.flay@oregonstate.edu

**Keywords:** Teens, smoking, tobacco, cessation, treatment, interventions

## Abstract

We have compared the unique features of teen tobacco cessation programs developed internally by community-based organizations (N=75) to prepackaged programs disseminated nationally (N=234) to expand our knowledge of treatment options for teen smokers. Internally-developed programs were more likely offered in response to the sponsoring organization’s initiative (OR=2.16, p<0.05); had fewer trained cessation counselors (OR=0.31, p<0.01); and were more likely found in urban areas (OR=2.89, p=0.01). Internally-developed programs more often provided other substance-abuse treatment services than prepackaged programs and addressed other youth-specific problem behaviors (p≤0.05). Studies that examine the effectiveness of internally-developed programs in reducing smoking and maintaining cessation for teen smokers are warranted.

## 1. Introduction

There are over 3.5 million smokers aged 18 and younger in the United States [1], and declines in teen smoking prevalence are stalling. Monitoring the Future data for 2007 show the past month smoking prevalence is 21.6% among 12^th^ graders [2]. With stalled declines prevailing, we are unlikely to achieve the Healthy People 2010 goal to reduce past month cigarette smoking among teens to 16% [3]. Estimates are that without accelerated cessation over 6.5 million teens alive today will ultimately die from smoking [4]. National surveys indicate high levels of motivation and attempts to quit smoking among teen smokers, but low success rates [5–7]. Most teens attempt to quit smoking without using treatment [8], even though there is evidence that behavioral treatment affords a two-fold increase in the likelihood of quitting [9].

Teen smoking cessation treatment (also referred to as programs or interventions) often consists of group-based counseling or behavioral interventions offered over the course of several sessions, and it mirrors the content and structure of adult cessation programs [10]. A recent meta-analysis that examined several types of cessation treatments, such as classroom-based efforts and clinic programs, found that the most effective teen smoking cessation treatment programs were multi-session, group-based programs with motivation enhancement, social influences, or cognitive-behavioral components that sought to identify and change thought processes that maintain use and teach skills to reduce use and promote cessation [9]. All of the treatment programs described in the meta-analysis were part of research studies. Although these treatment programs were effective for the small proportion of teens that attended, other intervention models also merit consideration. “Home-grown” programs or teen cessation programs developed internally by community-based organizations are one source of information about other models of intervention programming. Examining treatment programs developed by community-based organizations may provide insight about the local initiatives implemented to assist teens in quitting smoking [4]. Little is known about the characteristics of home- grown teen smoking cessation treatment, however. Using data from Helping Young Smokers Quit (HYSQ) Phase I, a national survey of community-based teen cessation programs [11], this paper describes the characteristics of teen smoking cessation treatment programs developed by community-based organizations and discusses their implications for research and practice.

### Background

Communities have been mobilized to reduce smoking among teens and have been at the center of several large, statewide comprehensive efforts to control tobacco use [12]. What is known about cessation efforts for teen smokers at the local community level is limited, but is growing. The first phase of HYSQ profiled a national sample of existing community-based tobacco cessation programs for teens in an effort to understand their prevalence. Of the 591 programs surveyed, Curry and colleagues [11] found that most (63%) of the community-based programs in the HYSQ sample used prepackaged teen smoking cessation treatments delivered in school-based settings to assist teens in quitting smoking. Prepackaged teen smoking cessation treatments are those that are developed and disseminated nationally by a variety of voluntary, governmental, or for-profit organizations, such as the American Lung Association and the American Cancer Society. Generally most prepackaged programs consist of voluntary multi-session, group-based efforts that are designed to reduce tobacco use and maintain cessation among teen smokers. These programs are designed to be delivered in a variety of settings, such as classrooms or schools and community youth groups. Most of the programs are available at-cost, are delivered by trained facilitators, and some have been recognized by federal agencies, such as the Centers of Disease Control and the Substance Abuse and Mental Health Services Administration, for their effectiveness in reducing tobacco use among teen smokers. Notably, 13% of the treatment programs in the HYSQ sample were developed solely by a community-based organization (also referred to as ‘internally-developed’ programs). Overall, Curry and colleagues concluded that the treatment programs used by the 591 participants in the sample were relatively homogenous and included many of the components that were found to be effective in the literature: most were school-based group treatments with multiple sessions that included state of the art cognitive-behavioral content.

Though the treatment programs profiled in HYSQ collectively had homogenous characteristics, an examination of internally-developed treatment programs is warranted. The current paper goes beyond that of Curry and colleagues by providing an in-depth examination of the local initiatives and programmatic efforts developed in applied community-based settings to assist teen smokers in quitting. Characterizing these programs may provide insight about the local communities’ perceived priorities regarding teen smoking cessation; about the organizational context in which the program was offered; and about the program implementation and delivery. Finally, closer examination of internally-developed programs may help us understand if what is known to be effective for teen smoking cessation in research settings is being practiced in community-based settings. The purpose of this paper is to characterize the unique features of internally-developed programs from several perspectives, including: characteristics of communities where these programs were found (e.g., in terms of urbanization, tobacco control prevalence, and the local perceptions regarding teen tobacco cessation); characteristics of the sponsoring organization (e.g., in terms of impetus of the program, staffing, funding, etc); and delivery and content of the programs. Phase I of HYSQ did not collect data on participant smoking cessation outcomes (i.e., quit rates). Though we are unable to determine if the internally-developed programs were efficacious for cessation, understanding the characteristics of internally-developed programs will expand our knowledge about available treatment options for teen smokers. To highlight the unique features of the internally-developed programs, we compare their community and organizational context, and program delivery and content to that of prepackaged programs.

## 2. Methods

### 2.1. Design

HYSQ is a multiphase, national initiative that addresses the critical need to disseminate effective, developmentally appropriate cessation programs for teen smokers. The first phase of HYSQ involved a national survey of existing community-based teen smoking cessation programs. Data for this paper come from that survey. Additional details regarding the design and sample process of the overall study can be found in Curry *et al.* [11].

### 2.2. Sample

A two-staged sampling design with United States counties as the first-stage probability sampling units was employed in this study. From this design, a total of 408 counties were selected to participate in the study. Next, snowball sampling was used in the 408 counties to identify administrators of tobacco cessation programs for teens. A total of 1,347 possible program administrators were identified through the snowball sampling procedure [11].

### 2.3. Program Eligibility

The 1,347 administrators were screened to determine eligibility as teen smoking cessation programs to be included in the study. A program was eligible if it was a teen smoking cessation program not a part of a research initiative that was established at least six months prior to the HYSQ evaluation and provided smoking cessation services to persons aged 12–24 years. Based on these criteria, 756 teen smoking cessation programs from 255 of the 408 counties were eligible to be included in the study.

### 2.4. Survey Procedures

Administrators of eligible teen smoking cessation programs were contacted and asked to participate in a 45-minute telephone interview that included questions about community context; organizational setting; participants; program delivery; program content and program evaluation. The telephone interview was administered by the University of Illinois at Chicago, Survey Research Laboratory. Program administrators received a paper copy of the survey prior to the telephone interview. A copy of this survey can be obtained at http://www.helpingyoungsmokersquit.org/.

Of the 756 eligible programs, program administrators from 591 teen smoking cessation programs completed the survey (78.2%). Reasons for not completing the survey included: respondents unavailable to complete the survey; respondents refused to take the survey; and/or respondents could not be reached after multiple attempts.

### 2.5. Measures

The telephone survey completed by program administrators included questions about community context; organizational setting; participants; program delivery; program content and program evaluation. No outcome measures (e.g., percentage of students who quit smoking) were included in this survey. Community context questions asked about the perceived concerns facing teens; local priorities regarding teen smoking cessation among community leaders; and program awareness and support of the teen smoking cessation program within the community. Organizational context questions (or characteristics of the sponsoring organization) asked program administrators about the primary reason for offering the teen smoking cessation program; the adequacy of funding for the organization; and staffing of the sponsoring organizations. Program content measures included questions about the inclusion of cessation strategies (e.g., smoking diaries, cigarette refusal skills, assessing nicotine dependence), content addressing other youth-specific issues (e.g., depression, academic performance, violence/gangs), youth treatment for other substances (e.g., alcohol, marijuana), medication use (e.g., nicotine patch, Zyban), use of incentives, and use of websites or quitline adjuncts. Program delivery measures included questions about the program operation (e.g., overall length and duration of the program), program format (e.g., group versus individual counseling), physical setting of the program, and enrollment criteria (e.g., voluntary, mandatory, or both). Participant recruitment and retention measures asked about the number of youth who were serviced by the program in the last 12 months and the number who completed the program in the last 12 months.

Measures of county stratification variables were also included. County stratification variables included county urbanization, socioeconomic status (SES), smoking prevalence, and per capita tobacco control expenditures. County urbanization was defined by U.S. Census Bureau metropolitan statistical area (MSA) and consisted of two categories: urban areas (MSAs) and rural areas (non-MSAs). SES was defined by federal poverty level, based on the 2000 Census data and had two categories: high SES (defined as state poverty level ≤ 20%) and low SES (defined as state poverty level > 20%). Smoking prevalence was defined by rates above the national median (31% for people aged 18–24, 2000 Behavioral Risk Factor Surveillance System) and was categorized as: high (defined as state prevalence > 31%) or not high (defined as state prevalence ≤ 31%). Tobacco control expenditures were categorized by tertiles into high, medium, and low expenditures.

### 2.6. Analysis

Prevalence of internally developed and prepackaged programs in the community strata was examined using chi-square tests. We investigated the bivariate associations of community context, organizational context, programmatic content, and implementation with internal and prepackaged programming. Next, we performed multivariate backward selection logistic regression to determine the best correlates of internal and external programming. Variables that were statistically significant (p≤0.05) at the bivariate level were included in the multivariate analyses. Analyses were conducted using SAS version 9.1.

## 3. Results

### 3.1. Participants

Of the 591 eligible programs that completed the survey, 374 (63.4%) reported that their organizations used smoking cessation materials developed by an external or parent organization (‘prepackaged programs’). When asked to name the specific prepackaged program used, just over half of the administrators (51.3%) reported using the American Lung Association’s Not-On-Tobacco (NOT) program. The two other prepackaged programs named most often were the Tobacco Education Group/Tobacco Awareness Program (TEG/TAP) (16.3%) and the “American Cancer Society” program (7.4%). The NOT [17] and TEG/TAP [18] programs have been examined in the literature for their effectiveness in promoting short-term smoking cessation and cigarette reduction among teens. State-based prepackaged programs comprised the remaining 25%. Of those who used external programming, 234 organizations reported implementing their programs ‘very closely’ to the design or specifications of the external organization; 131 implemented ‘somewhat closely’; and nine implemented not very closely. Seventy-five (12.7%) organizations reported developing their own teen smoking cessation materials internally (‘internally developed’). The remaining 141 (23.9%) reported using both internally-developed and prepackaged materials. This paper describes data from the 234 organizations that reported implementing prepackaged programs “very closely” and from the 75 organizations that reported using exclusively internally-developed programs (total n=309 organizations). Our decision to select only the 234 prepackaged programs was based on our interest in comparing the unique features of the internally-developed programs to those prepackaged programs that had very few modifications.

#### Community context

Table 1 presents the bivariate associations for the characteristics of the community and the organizations that sponsored the internally developed and prepackaged programs. Internally-developed programs were less prevalent in rural (non-MSA) areas and in areas with high smoking prevalence. No significant differences were found for other community characteristics, including concerns facing teens, community leaders’ level of priority for teen tobacco cessation, and the general population’s awareness and support of teen tobacco cessation programs.

### 3.2. Sponsoring Organizational Context

Compared to those using prepackaged programs, organizations that developed their own cessation program internally reported that their program was more likely to be offered in response to the initiative of the organization. Those organizations that developed their own treatment programs internally were less likely to report having adequate sources of funding. They were also less likely to have counselors trained specifically in teen smoking cessation, and more likely to report program delivery by physicians and certified health educators compared to those organizations using prepackaged programs. On average, both organizations using internally-developed programs and those using prepackaged programs had one paid full-time equivalent (FTE) of staff working with the program.

Administrators of both internally-developed programs and prepackaged programs reported that obtaining sufficient enrollment of participants was very challenging; those using prepackaged programs were more likely to endorse this than those using internally-developed programs. Other challenges experienced by both included: obtaining follow-up information on participants, obtaining sufficient funding, hiring appropriate staff for the program, and keeping participants in the program. Despite these challenges, about three-fourths of administrators from both internally-developed and prepackaged programs indicated they were “very likely” to be operating in one year.

### 3.3. Program Delivery

Table 2 describes the program delivery and content of the internally-developed programs and prepackaged programs. The majority of internally-developed and prepackaged programs were school-based and offered treatment in group-based settings. There were no significant differences between programs with regard to length and duration of treatment. Internally-developed programs were more likely than prepackaged programs to have mandatory enrollment criteria. Internally-developed programs were less likely than prepackaged programs to include a written manual or guide for the program and to conduct evaluation.

### 3.4. Program Content

There were several differences in program content between internally-developed and prepackaged programs. Internally-developed programs were more likely than prepackaged programs to address other youth-related issues, such as other drug use, life goals (e.g., employment), and violence or gangs. Similar to prepackaged programs, internally-developed programs included state of the art cognitive-behavioral strategies, such as self-monitoring, disrupting smoking patterns, and coping skills training. These programs were also less likely than prepackaged programs to offer incentives overall to participants.

### 3.5. Multivariate Analyses

Characteristics that distinguished internal and prepackaged programs in the bivariate analyses were entered into a multiple logistic regression model to determine the best correlates of cessation programming. Of the 234 prepackaged programs and 75 internally-developed programs, 58 observations from the prepackaged programs and 10 observations from the internally-developed programs were removed from the analysis due to missing values for the responses or the explanatory variables (68/309 or 22% loss). The analysis was conducted on the remaining 176 prepackaged programs and the 65 internally-developed programs. Results are presented in Table 3.

State-level smoking prevalence no longer significantly distinguished internal and prepackaged cessation programming in the multivariate model. County urbanization remained a significant correlate of cessation programming, with internally-developed programs more likely to be found in urban (MSA) areas. Similar to the bivariate analyses, the multivariate analyses indicated that organizational characteristics such as offering a program in response to an initiative of the organization, having counselors trained in cessation, and having physicians on the program’s staff remained significant correlates of cessation programming; including certified health educators and enrollment challenges were no longer significant correlates.

Bivariate analyses suggested that the content of internally-developed programs more often included topics related to other youth development issues and also included recommended state-of-the-art cessation strategies. Addressing other youth-related issues was no longer a significant correlate of cessation programming in the multivariate model, however. Only the cessation strategy, keeping diaries of smoking, was a significant correlate of cessation programming. No program delivery characteristics were significant correlates of cessation programming in the multivariate model.

## 4. Discussion

The purpose of this paper was to describe the characteristics of internally-developed programs, compared to prepackaged programs, specifically examining the community and organizational contexts in which the programs were offered, and the delivery and content of the smoking cessation programming itself. Compared to prepackaged programs, our findings suggest that there were some unique features of the internally-developed programs with regard to the community and organizational contexts in which they were offered, as well as in the content of the programs.

Internally-developed programs were more often found in urban communities. Our data precluded an explanation of why these programs appear to be more prevalent in urban communities, however. As might be expected, internally-developed programs were more likely created in response to the initiative of their sponsoring organization than in response to state legislation or a health department or department of education initiative. Although the data did not permit us to examine additional reasons why some organizations chose to develop their own programs in light of available prepackaged programs, our findings allowed us to speculate possible scenarios. Organizations that developed their own cessation programs internally were more likely to report fewer resources, such as fewer trained cessation counselors, compared to those using prepackaged programs. Perhaps fewer resources affected the ability of some organizations to obtain and fully implement prepackaged programs. Some sponsoring organizations also may have developed their own programs to better suit the needs of their participants. Bivariate analyses suggested that internally-developed programs were more likely to address other youth-related issues, such as other drug use, life goals, and violence or gangs. Perhaps organizations that developed cessation programming internally served high-risk teens who were dealing with other youth-related problem behaviors and issues in addition to tobacco use. That these programs included content on many health issues may suggest that less tobacco cessation content was provided to teens, compared to prepackaged programs. Continued investigations regarding why some chose to develop their own cessation programming internally and if less cessation content was provided to teens warrants further study.

Other unique features of internally-developed programs include criteria for enrollment in the program, the programmatic content itself, and the presence of physician staff. Although mandated participation is relatively uncommon in research-based teen smoking cessation treatment programs [13,14], it was more prevalent among internally-developed programs. As noted earlier, internally-developed programs covered youth-specific issues beyond what is usually addressed in prepackaged cessation programs, such as career planning, violence/gangs, and other drug use. Topics related to developmental issues and other problem behaviors may have been included to address the needs of teens that may have been at risk for engaging in multiple problem behaviors, which could be typical of teens who are mandated to participate in teen smoking cessation treatment programs. In a qualitative study of minors cited for possession of tobacco, Loukas and colleagues [15] reported that some of the teens mandated to attend a tobacco awareness class were involved in other problem behaviors, such as alcohol or other drug use. That the internally-developed programs were more likely to be staffed by physicians than prepackaged programs may imply that these practitioners believed they had the requisite knowledge to develop their own programs and address the needs of their participants.

Though unique features were present, there were a number of similarities between internally-developed and prepackaged programs. With regard to community and organizational context, reported community priority and support for teen tobacco control was similar among organizations using either internally-developed or prepackaged programs. Organizations that reported using either program type reported similar challenges, such as obtaining sufficient enrollment. There were also similarities with regard to program delivery and content. Similar to prepackaged programs, our results indicated that internally-developed programs utilized programmatic strategies known to be effective in reducing smoking among teens. For instance, internally-developed programs were offered in multiple-session groups in school-based settings, similar to prepackaged programs. Likewise, internally-developed programs in our sample included cognitive-behavioral cessation components in their content, though to a lesser degree than the prepackaged programs. Current youth smoking cessation guidelines recommends the use of cognitive-behavioral treatment approaches to help teen smokers quit [10, 13] and suggests offering treatment in multiple sessions [9]. In a report of best practices for teen smoking cessation practice, Orleans and colleagues [16] noted the importance of disseminating evidence-based findings to widespread applications beyond research settings. That internally-developed programs show consistency in implementing some of the evidence-based strategies is encouraging and implies that local community-based organizations that develop their own cessation programming internally are aware of the current state-of-the-art treatment components and are incorporating them in practice.

Although the current study extends our knowledge regarding the characteristics of internally-developed programs, there are limitations. As noted earlier, the survey did not assess tobacco use or smoking cessation outcomes of the participants. As reported by Curry and colleagues [11], the aim of the first phase of HYSQ was to describe a national sample of available teen tobacco cessation programs through information obtained by program administrators. The study did not collect any data from program participants so we were unable to determine if the internally-developed or prepackaged programs in our sample were efficacious for teen smoking cessation. Second, the comparison group of prepackaged programs included data only from organizations that reported ‘very close’ program adherence. Also, our findings are based on a small sample of internally-developed programs. Taken together, the results of this study may not be representative of all internally-developed or prepackaged programs. Finally, data from the program administrators were self-reported data and were not validated by any other source.

In conclusion, our findings suggest that there are unique features of teen smoking cessation programs developed internally by local community-based organizations. These findings have several implications for practice and research. Internally-developed programs incorporated some state-of-the-art efficacious components into their treatment, implying that evidence-based research practices are being applied in practice settings. Program providers in local communities that have the support of their sponsoring organization should be encouraged to develop cessation programming that includes these efficacious components and evaluate them as implemented to determine program efficacy. To facilitate the adoption of effective practices in community settings, there is a need for program developers and researchers to collaborate to develop cessation programming. Researchers can provide training and technical assistance in the development of evidence-based treatments to community-based program providers who are interested in developing their own programs to meet the needs of their participants and their sponsoring organizations. Research studies are needed to obtain representative samples of internally-developed programs to further examine their impetus for development. For instance, these studies may examine the ability and willingness of community-based program developers to implement prepackaged programs. Studies are also needed to examine the impact of these programs on teen smoking cessation outcomes (e.g., percentage of quit rates, reduction in smoking), particularly the ability of these programs to either enhance or result in a loss of effectiveness compared to prepackaged programming.

## Figures and Tables

**Table 1.** Community and organizational context of internally-developed and prepackaged programs.

	Internally developed (N = 75, %)	Prepackaged programs (N = 234, %)	χ^2^ or F
**Community context**^1^			
County stratification			
In a rural (Non-MSA) area	18.7	34.3	6.57**
In a low SES areas	10.7	15.5	1.06
In an area with high smoking prevalence	38.7	53.2	4.81*
In an area with high tobacco control expenditures	41.3	49.4	1.64

Biggest concern facing youth			0.01

Other & drug use, not including tobacco 2	81.3	81.7	
Tobacco/Drug use & Tobacco/No drug use	18.7	18.3	

Community leader priority on youth tobacco cessation			0.06

A high priority	17.3	18.5	
Somewhat of a priority	65.3	64.2	
Not a priority at all	17.4	17.3	

Awareness of general population of tobacco cessation program			2.68

Very aware	10.7	5.6	
Somewhat aware	70.7	71.2	
Not at all aware	18.6	23.2	

General population’s support of tobacco cessation program			1.30

Very supportive	65.6	59.2	
Somewhat supportive	34.4	39.7	
Not at all supportive	0.0	1.1	
**Organizational context**			
Primary reason for offering the program			
Initiative of the organization	52.7	35.1	7.31**
Other reasons 3	47.3	64.9	
Program funding			
Adequacy of funding (scale 1–5)	3.13	3.61	–2.64**
Program staffing			
Counselors trained specifically in smoking cessation	77.1	91.8	11.19**
Professional background of staff 4			
Physician	13.3	3.4	10.11**
Nurse	40.0	36.9	0.23
Dental professional	4.0	3.4	0.05
Teacher	40.0	41.2	0.03
Coach	18.7	16.3	0.22
Social worker	29.3	27.5	0.10
School counselor	38.7	36.5	0.12
Certified health educator	49.3	35.9	4.27*
Trained tobacco counselor	62.2	58.6	0.29
Youth peer	17.8	16.4	0.08
Challenges			
Sufficient enrollment	24.0	37.1	6.46*
Hiring appropriate staff	23.0	13.4	4.17
Retaining hired staff	14.7	9.1	2.72
Recruiting staff volunteers	16.0	16.4	2.34
Keeping participants in program	22.7	12.5	5.10
Obtain follow-up information	32.4	22.6	3.43
Obtain sufficient funding	23.6	18.3	1.25

Likelihood of operating in 1 year	72.0	75.8	1.96

* *p*≤.05;

** *p*≤.01;

*** *p*≤.001

^1^ Cell percentages may not total 100% for all characteristics. Participants had the option to select from several response options.

^2^ Categories were collapsed because too few numbers were in the cells. Categories were 1. Other, 2. Drug use, no tobacco, 3. Tobacco and Drug use (above as Tobacco/Drug Use), 4. Tobacco, no drug use (above as Tobacco/No drug use)

^3^ Categories were collapsed because too few numbers were in the cells. Other reasons included 1. Legislation with penalty for youth possession, use and/or purchase of tobacco; 2. A response to the health department or department of education initiative or mandate; 3. Youth demand; 4. Parent demand; 5. School/teacher demand; 6. Something else.

^4^ Column percentages do not total to 100%. Respondents were asked to describe the professional backgrounds of the program staff involved in the direct provision of services to participants and say “yes or no” for each. The responses represent the percent who responded yes for each profession.

**Table 2.** Implementation and content of internally-developed and prepackaged programs.

Characteristic 1	Internally developed (N=75, %)	Prepackaged programs (N=234, %)	χ^2^ or F
**Program Delivery**			
Program format			
Face to face	65.3	53.7	3.13
Group	85.3	97.9	18.06***
Phone counseling	21.9	14.3	2.39
Internet	5.4	7.3	0.32
Self-help manuals	54.7	44.2	2.51
Physical setting			
Community center	18.4	16.1	0.13
Faith-based organization	14.3	10.6	0.47
School-based setting	81.3	90.6	4.68*
Drug treatment center	12.5	11.6	0.03
Sports/health club	4.1	9.8	1.54
Program operation			
Program length (number of contacts)			–1.30
Mean, S.D Median	7.5 (7.3) 6	8.7 (4.2) 10	
Program duration (days)			–0.56
Mean, S.D. Median	66.3 (74.4) 49	72.1 (73.7) 63	

Enrollment criteria			8.78**

Voluntary	44.0	62.1	
Mandatory	17.3	8.6	

Number of participants in the past 12 months prior to the survey			–0.90
Mean, S.D Median	83.5(133.8) 31	325.2 (4000) 20	

Possess written facilitator guide or manual	72.0	95.7	35.23***

Includes an evaluation component	67.6	82.1	7.01**
**Program Content**			
Address other youth-related issues			
Depression	62.7	52.8	2.22
Self-esteem	85.3	80.1	1.03
Stress	92.0	95.7	1.58
Academic performance	61.6	52.2	2.02
Violence or gangs	28.8	17.6	4.29*
Employment	36.5	21.7	6.54**
Career planning	33.3	16.1	10.39***
Other drug use	74.0	56.5	7.13**
Alcohol	71.6	48.1	12.53***
Inclusion of cessation strategies			
Keep diaries of smoking	61.1	87.2	24.16***
Practice refusing cigarette offers	83.8	92.7	5.22*
Sign a contract that has rewards for not smoking	35.6	58.2	11.25***
Sign a contract that has penalties for smoking	13.5	6.0	4.34*
Invite a family member to participate	52.1	37.7	4.69*
Assess level of nicotine dependence	84.9	89.7	1.22
Practice ways of coping with temptations	97.3	98.3	0.26
Do any of aversive smoking	4.0	4.7	0.07
Throw away all of smoking-related paraphernalia	67.1	69.6	0.16
Practice meditation	87.8	90.5	0.44
Change diet in any way	66.2	72.7	1.14
Increase physical activity	84.0	90.1	2.08
Gradually reduce or taper smoking	83.8	82.3	0.09
Change cigarette brands	17.6	24.5	1.51
Identify specific people to help quit	93.3	91.9	0.17
Speak to younger children about not smoking	43.2	33.2	2.47
Invite a peer/friend to participate	61.3	61.2	0.00

Provide incentives	35.1	59.8	13.72***

Gift certificates 2	53.9	24.3	9.27**
Leave time from class 2	46.2	75.7	19.84***

* *p*≤.05;

** *p*≤.01;

*** *p*≤.001

^1^ Cell percentages may not total 100% for all characteristics. Participants had the option to select several response options.

^2^ Specific types of incentives offered, as indicated by those who responded that they provided any incentives to their participants.

**Table 3.** Multivariate model for internally-developed and prepackaged programs.

	B	SE	*P*	OR	CI (95%)
MSA vs. non-MSA	1.05	0.42	0.01	2.89	1.25–6.58
High SES vs. Low SES	0.56	0.55	0.31	1.74	0.60–5.11
High smoking prevalence vs. low smoking prevalence	–0.36	0.34	0.28	0.70	0.36–1.34
High expenditures vs. low expenditures	0.00	0.43	0.98	1.00	0.44–2.32
Medium expenditures vs. low expenditures	0.13	0.46	0.77	1.14	0.47–2.78
Developed in response to organizational leadership vs. other	0.77	0.33	0.02	2.16	1.13–4.13
Keep diaries of smoking	–1.34	0.38	<0.001	0.26	0.13–0.55
Use of incentives	–1.00	0.34	<0.01	0.37	0.19–0.72
Use of trained counselors	–1.15	0.44	<0.01	0.31	0.13–0.74
Physician staff	1.58	0.64	0.01	4.85	1.37–17.10
